# Sexual health literacy level and its related factors among married medical sciences college students in an Iranian setting: a web‑based cross‑sectional study

**DOI:** 10.1186/s12978-024-01756-7

**Published:** 2024-04-17

**Authors:** Samaneh Nematzadeh, Zohreh Shahhosseini, Mahmood Moosazadeh, Zeinab Hamzehgardeshi

**Affiliations:** 1 Department of Midwifery Counseling, Student Research Committee, Mazandaran University of Medical Sciences, Sari, Iran; 2 Department of Midwifery, School of Nursing and Midwifery, Sexual and Reproductive Health Research Center, Mazandaran University of Medical Sciences, Sari, Iran; 3 Gastrointestinal Cancer Research Center, Noncommunicable Diseases Institute, Mazandaran University of Medical Sciences, Sari, Iran

**Keywords:** Sexual health, Literacy level, Medical students, Iran

## Abstract

**Background:**

Sexual health literacy (SHL) leads to the development of personal ability, understanding, evaluation and use of information related to sexual health. The purpose of this study was to assess the sexual health literacy level and its related factors among married college students at Mazandaran University of Medical Sciences (MAZUMS).

**Methods:**

A web-based cross-sectional online study was conducted on married college students at Mazandaran University of Medical Sciences between January and November 2020. All students were included in the study by census, and the study method was explained by telephone. If they agreed to participate in the study, the online link to the questionnaire, including sociodemographic and clinical information and Sexual Health Literacy for Iranian Adults (SHELIA), was emailed. The Statistical Package for the Social Sciences (SPSS) software version 26 was used for data analysis. Univariate and multivariate logistic regression tests were used to assess factors related to sexual health literacy.

**Results:**

The sample consisted of 277 male and 123 female students. Sexual Health Literacy Level and all subscales are at the sufficient level (66.1–88). Among the participants, 20.5% had limited sexual health literacy. Multivariate analysis found factors related to sexual health literacy among students: economic status (OR 0.03; 95% CI 0.0–0.55) and faculty (OR 0.07; 95% CI 0.01–0.52) is related to decrease and subscription to social media for sexual health (OR 3.27; 95% CI 1.53–7.01), information source of channels and cyberspace (OR 3.23; 95% CI 1.41–7.39), educational level (OR 16.39; 95% CI 2.16–32.70), Internet search information source (OR 1.91; 95% CI 1.00–3.64) is related to increase, were statistically significant factors.

**Conclusion:**

In Iran, medical sciences college students, who constitute a significant portion of the country's population, are responsible for sexual health education. Government agencies, with the collaboration of all stakeholders, should develop policies and programs for implementing and evaluating integrated and comprehensive sexual health literacy promotion programs for them.

**Supplementary Information:**

The online version contains supplementary material available at 10.1186/s12978-024-01756-7.

## Background

As per the definition by the World Health Organization (WHO), health literacy encompasses an individual's capacity to access, comprehend, interpret, and utilize health-related information essential for making well-informed decisions in the realm of health [1]. Among the various dimensions of health, sexual health holds a significant position and serves as a crucial indicator of community well-being. Sexual health literacy (SHL) pertains to a diverse range of competencies in the domain of sexual health. These competencies encompass areas such as sexual development, puberty, pregnancy, contraceptive methods, unintended pregnancies, sexually transmitted diseases, acquiring skills to manage sexual relationships—including discussions about preferences and boundaries—and recognizing the positive and romantic aspects of these relationships [2, 3]. A robust level of sexual health literacy equips individuals to critically assess, make decisions about, and modify their sexual behaviors, thereby facilitating the nurturing, upkeep, and advancement of sexual well-being [4]. In simpler terms, sexual health literacy fosters a deeper comprehension and evaluation of risks related to sexual health, leading to safer sexual encounters, diminished instances of unintended pregnancies and infections, and enhanced family and societal health [5, 6].

Recent findings reveal concerning statistics: in the UK, 41.6% of men and 51.2% of women report experiencing at least one type of sexual problem [7]. Similarly, in Iran, a meta-analysis demonstrated a prevalence of sexual dysfunction among women of 52% [8]. Moreover, unwanted pregnancies have been reported globally, with a prevalence rate of 44% [9]. In Iran, a meta-analysis pinpointed the prevalence of unwanted pregnancies at 52% [10]. The Centers for Disease Control and Prevention has alarming statistics—46% of individuals aged 15 to 24 did not use condoms during their most recent sexual encounter. This group accounted for 21% of new HIV diagnoses and over 50% of new cases of other sexually transmitted infections [11]. Notably, even in Iran, 15.7% of medical students exhibit risky behaviors, including those of a sexual nature [12]. This is particularly concerning given the looming risk of a renewed wave of HIV infections through sexual contact [13]. Research findings consistently demonstrate that individuals, particularly young ones, with lower levels of sexual health literacy exhibit reduced condom use, engage in riskier sexual behaviors, and face heightened odds of unintended pregnancies [14–16].

The scale of sexual health literacy among different populations and countries was diverse. Scale for measuring sexual and reproductive health literacy of adolescents in Lao PDR was a self-administered structured questionnaire including five parts: (1) Socio-demographic, (2) Personal health-lifestyle, (3) SRH knowledge & behavior, (4) SRH literacy and (5) Functional literacy on condoms. The sexual and reproductive health literacy score included 4 levels: inadequate, problematic, sufficient and excellent. Both the formula and the scale are adopted from the European Health Literacy Survey method [17]. Scale to measure women's sexual and reproductive health literacy in Armenia, valid surveys in Armenian language, which questions included demographic information and risk factors, symptoms and prevention methods of STI and cervical cancer, as well as contraceptive options for women [18]. Scale for measuring the sexual health literacy of students in Australia was used from the total scores of two questionnaires, which include the questionnaire of the Australian Research Center includes knowledge and HIV/Hepatitis domains and University of Missouri Sexual Health Survey includes knowledge, STI and pregnancy domains [17].

It is often assumed that university students possess the life skills necessary, including knowledge about sexual health and sexuality, to navigate their academic journey and beyond. However, evidence points to an uneven distribution of sexual health literacy, with certain groups lagging behind [17]. The significance of medical and nursing students' specialized involvement lies in their role, not only in safeguarding their own health but also in caring for future patients. Surprisingly, numerous medical and nursing students lack adequate sexual health literacy [18, 19], potentially due to providers' reluctance to discuss sexual matters with patients and their inclination to impose personal values on patient care [20, 21].

Diverse factors are interlinked with sexual health literacy. These factors can be categorized into demographic, sociocultural, and medical-fertility domains. Demographic factors encompass gender, age, marital status, education, race, location of residence and study, and field of study [16, 17, 22–26]. Sociocultural factors include religion, economic standing, knowledge related to sexual health and fertility, participation in sexual education programs, sexual experience, and sources of information [5, 16, 17, 26, 27]. Meanwhile, medical-fertility factors, such as contraceptive usage, familiarity with condom application, and single or multiple pregnancies, are closely linked to the level of sexual health literacy [25, 26, 28]. Notably, enhancing sexual health literacy among young individuals is a potent strategy for promoting positive sexual health behaviors [20].

Despite a plethora of available information on sexual health literacy, few comprehensive national or regional studies have been conducted in Iran that encompass all facets of sexual health literacy and beliefs [24, 25]. Intriguingly, there has been no investigation into sexual health literacy specifically among student populations. Given that incorrect or insufficient information about sexual health has far-reaching effects on reproductive health and marital life, gauging the sexual health literacy of this demographic becomes paramount for crafting effective health promotion initiatives. Discrepancies in data across various realms of sexual health literacy have been noted in global studies. Thus, the primary objective of this cross-sectional study is to assess sexual health literacy and its determinants among married college students at Mazandaran University of Medical Sciences (MAZUMS).

## Methods

### Research design and participant selection

This study employed a web-based cross-sectional approach conducted online from June to November 2020. The target participants were married students at Mazandaran University of Medical Sciences who held bachelor's, master's, general doctorate, or Ph.D. degrees. The inclusion criteria encompassed all married students with internet accessibility. Participant recruitment involved reaching out to all eligible married students through a census approach, with the study's methodology explained via telephone communication. Upon agreement to participate, married students received links to the online questionnaires, which were disseminated using social media platforms or email. The contact details of married students were sourced from the university's cultural department. Notably, participation in the study was entirely voluntary and anonymous. Participants received no incentives or compensation for their involvement.

### Procedure

The online questionnaire was designed in Persian and consisted of closed-ended questions. The questionnaire was created using Google Forms, and the questionnaire link was shared through social media channels or email. Ethical approval was secured from the Biomedical Research Ethics Committee of MAZUMS (Approval code: IR.MAZUMS.REC.1399.364). All research procedures adhered to the prescribed research plan. Furthermore, the reporting and preparation of this article followed the guidelines laid out in the STrengthening the Reporting of OBservational studies in Epidemiology (STROBE) statement checklist (Additional file 1).

### Questionnaire structure

The questionnaire encompassed two distinct sections (Additional files 2, 3). The initial section aimed to capture sociodemographic and clinical particulars, which encompassed variables such as age range, gender, residential area, educational attainment, faculty affiliation, spouse's educational background, religious affiliation, occupational status, marriage duration, sources of sexual information, completion of family knowledge courses, economic standing, student living arrangements, participation in sexual health workshops, premarital sexual experiences, subscription to sexual health-related content on social media, access to media and the internet, utilization of contraceptive methods, specific type of contraceptive used, condom utilization, history of abortion, occurrences of sexual abuse, and history of sexually transmitted diseases (STDs).

The second section of the questionnaire introduced the "Sexual Health Literacy for Iranian Adults" (SHELIA) scale [29]. This scale, developed and validated in Iran, comprises 40 questions and primarily serves to gauge the sexual health literacy of Iranian adults. It encompasses four dimensions: access skills (8 items), reading and comprehension skills (17 items), analysis and evaluation skills (5 items), and application skills (10 items), which collectively account for 68.1% of the variance. Respondents rated their responses on a 5-point Likert scale, attributing scores ranging from one for "strongly disagree" to five for "strongly agree". Subscale scores were derived through summation of individual responses. A conversion formula was subsequently employed to transform raw scores into a scale that spans from zero to 100**..**$$\frac{Score obtained - minimum raw score}{Maximum Score - Minimum Score}\times 100$$

Calculation of the overall questionnaire score involves averaging the scores across the various domains of sexual health literacy. The scores on the questionnaire were categorized as follows: inadequate (0–50), acceptable (50.1–66), sufficient (66.1–84), and excellent (84.1–100). The content validity of the items was established with a content validity index (CVI) of 0.84 and a content validity ratio (CVR) of 0.81. Exploratory factor analysis identified four mentioned Dimentions that explained %68.1 of the variance. Convergent validity of the questionnaire showed a correlation between the questionnaire’s dimensions and general health literacy questionnaire in the range of 0.31 to 0.70. The SHELIA questionnaire demonstrated strong internal consistency reliability, with Cronbach's alpha ranging from 0.84 to 0.94 and intraclass correlation coefficients (ICCs) ranging from 0.90 to 0.97 [29]. The author of the questionnaire, Dr. Maasoumi, granted authorization for its use in this study.

### Statistical analysis

Data analysis was conducted using the Statistical Package for the Social Sciences (SPSS) software version 26. The study population consisted of 480 married students, with 400 completing the questionnaires based on information from MAZUMS' cultural deputy. Descriptive statistics included the mean ± standard deviation (SD) for quantitative data and frequency along with percentage for qualitative data. The normality of variables was assessed using the Kolmogorov‒Smirnov and Shapiro‒Wilk tests. Comparative analysis employed Mann‒Whitney U tests for two-group comparisons and Kruskal‒Wallis tests for comparisons involving more than two groups. Univariate and multivariate logistic regression analyses were employed to explore factors associated with sexual health literacy. For independent variables with more than two categories, dummy variables were utilized. A significance level of p < 0.05 was considered statistically significant.

## Results

Of the 400 participants enrolled in the study, 277 were male, and 123 were female. The age distribution showed that 39% fell within the 15–25 age bracket, while 37.5% belonged to the 26–35 age range. Half of the participants were pursuing their undergraduate education, with 61.7% of students reflecting a moderate economic status. Detailed sociodemographic and other participant characteristics are presented in Table 1.

**Table 1:** Sociodemographic and clinical information of the studied sample (N = 400)

Variables		Frequency (%)	SHL Mean ± SD
Age range	15–25	156 (39.0%)	74.86 ± 13.17
	26–35	151 (37.7%)	76.99 ± 12.15
	36–45	76 (19.0%)	73.99 ± 12.07
	> 45	17 (4.3%)	77.14 ± 10.41
Gender	Female	123 (30.8%)	74.50 ± 12.80
	Male	277 (69.2%)	76.08 ± 12.35
Living area	City	374 (93.5%)	75.80 ± 12.49
	Urban	26 (6.5%)	72.73 ± 12.49
Educational level	Bachelor’s degree	203 (51.0%)	73.66 ± 12.41
	Master degree	113 (28.0%)	78.27 ± 12.64
	General doctorate	61 (15.0%)	77.31 ± 12.06
	Ph.D	23 (6.0%)	75.48 ± 9.95
Faculty	Medicine	59 (14.8%)	76.69 ± 11.61
	Dentistry	23 (5.8%)	71.01 ± 12.32
	Pharmacy	17 (4.2%)	80.12 ± 8.86
	Health	95 (23.8%)	74.79 ± 11.88
	Nursing and midwifery	107 (26.8%)	79.74 ± 13.78
	Paramedical	96 (24.0%)	71.53 ± 11.19
	New technologies	3 (0.8%)	71.06 ± 7.23
Spouse's education level	High school and diploma	26 (6.5%)	76.59 ± 13.39
	Bachelor’s degree	244 (61.0%)	75.03 ± 12.27
	Master degree	64 (16.0%)	77.94 ± 12.99
	General doctorate	43 (10.7%)	75.28 ± 12.44
	Ph.D	13 (3.3%)	77.45 ± 12.31
Religion	Islam	397 (99.2%)	75.69 ± 12.29
	Other*	3 (0.8%)	62.92 ± 31.67
job	With a fixed salary	178 (44.5%)	75.13 ± 11.39
	No fixed salary	37 (9.3%)	77.31 ± 15.06
	Student	185 (46.2%)	75.70 ± 12.97
Duration of marriage (year)	Less than 1	60 (15.0%)	75.71 ± 14.24
	1–3	121 (30.3%)	75.72 ± 11.24
	3–5	50 (12.4%)	75.52 ± 15.25
	More than 5	169 (42.3%)	75.50 ± 11.90
Sexual Information Source	Friends	72 (18.0%)	72.85 ± 10.40
	Parents	14 (3.5%)	72.76 ± 11.08
	Sister and brother	10 (2.5%)	71.52 ± 7.61
	Relatives	7 (1.8%)	81.10 ± 12.47
	Internet	258 (64.5%)	75.51 ± 13.20
	Channels and cyberspace	90 (22.5%)	74.47 ± 11.44
	Media	35 (8.8%)	75.89 ± 14.74
	Health care providers	156 (39.0%)	78.66 ± 11.71
Passing family knowledge course	Yes	330 (82.5%)	76.50 ± 12.08
	No	70 (17.5%)	71.34 ± 13.55
Economic status	Weak	8 (2.0%)	65.22 ± 16.26
	Medium	247 (61.7%)	75.19 ± 12.12
	Good	135 (33.8%)	76.73 ± 12.29
	Excellent	10 (2.5%)	78.49 ± 17.89
Student's residence	Dormitory	345 (86.2%)	76.03 ± 12.75
	Private house	55 (13.8%)	72.85 ± 10.43
Participation in sexual health workshop	Yes	77 (19.3%)	80.31 ± 11.91
	No	323 (80.7%)	74.47 ± 12.38
Premarital sexual experience	Yes	44 (11.0%)	76.97 ± 15.16
	No	356 (89.0%)	75.42 ± 12.13
Subscribe to social media** for sexual health	Yes	173 (43.3%)	77.82 ± 12.37
	No	277 (56.7%)	73.90 ± 12.34
Media and Internet access	Yes	398 (99.5%)	75.67 ± 12.48
	No	2 (0.5%)	61.32 ± 3.86
Use of contraceptive methods	Yes	285 (71.3%)	76.90 ± 12.11
	No	115 (28.7%)	72.37 ± 12.89
Type of contraception	Natural methods	161 (40.2%)	75.61 ± 11.85
	Condom	145 (36.2%)	75.61 ± 12.22
	Tablet	24 (6.0%)	79.26 ± 14.51
	Injection	1 (0.3%)	80.11 ± 0.00
	Intrauterine devices	8 (2.0%)	75.99 ± 10.9
	Surgery	40 (10.0%)	71.11 ± 14.76
	None	21 (5.3%)	79.41 ± 11.63
Use of condom	Always	95 (23.8%)	76.66 ± 13.44
	Sometimes	147 (36.7%)	77.55 ± 11.46
	Usually, not	59 (14.8%)	73.77 ± 13.95
	Never	99 (24.7%)	72.76 ± 11.60
Abortion	Never	161 (40.3%)	76.47 ± 12.19
	Once	215 (53.7%)	74.74 ± 12.19
	More than once	24 (6.0%)	77.47 ± 10.32
History of sexual abuse	Yes	13 (3.3%)	77.71 ± 10.07
	No	387 (96.7%)	75.52 ± 12.57
History of sexually transmitted diseases	Yes	47 (11.8%)	74.76 ± 11.85
	No	353 (88.2%)	75.70 ± 12.59
	Total	400 (100.0%)	

*Other religions: Christian and Zoroastrian; **Social media: Telegram, Instagram, and WhatsApp

Given the study's categorization of insufficient and acceptable levels as low, these two levels were merged. Similarly, sufficient and excellent levels were combined. Consequently, sexual health literacy was classified into a two-tier variable representing poor and appropriate levels.

The average cumulative score for sexual health literacy was calculated at 75.59 ± 12.49. All subscales were observed to be within the adequate range (66.1–88), with the Reading and Comprehension skills displaying the highest mean score of 80.60 ± 13.12 among the SHELIA subscales (Table 2).

**Table 2:** Sexual health literacy score and its subscales

Subscales	Minimum	Maximum	Mean	SD
Accessibility skills	25	100	76.35	16.14
Reading and comprehension skills	44.44	100	80.60	13.12
Analysis and evaluation skills	25	100	70.60	17.47
Application skills	25	100	74.83	13.41
Total	36.74	100	75.59	12.49

### Univariate analysis to assess factors related to sexual health literacy

The univariate analysis revealed notable trends among the student population. Those with poor economic status (with a distribution of 75% for poor SHL and 25% for appropriate SHL) exhibited decreased odds of possessing sexual health literacy (OR = 0.04, 95% CI: 0.0–0.51) compared to their counterparts with excellent economic status (with percentages of 10% for poor SHL and 90% for appropriate SHL). Likewise, students who did not pass the family knowledge course (with proportions of 34.2% for poor SHL and 65.8% for appropriate SHL) displayed diminished odds of having sexual health literacy (OR = 0.40, 95% CI: 0.23–0.71) in comparison to those who successfully completed the family knowledge course (with 17.2% for poor SHL and 82.8% for appropriate SHL) (Table 3).

**Table 3:** Logistic univariate and multivariate analyses showing the association of sociodemographic and clinical information with sexual health literacy

Variables		N	Poor SHL n = (%)	Appropriate SHL n = (%)	Crude OR (95%CI)	p value	Adjusted OR (95% CI)	p value
Living area	City	374 (93.5%)	73 (19.5%)	301 (80.5%)	1.83 (0.77,4.38)	0.173	1.21 (0.42, 3.50)	0.721
	Urban	26 (6.5%)	8 (30.8%)	18 (69.2%)	Reference		Reference	
Economic status	Poor	8(2.0%)	6 (75%)	2 (25%)	0.04 (0.0, 0.51)	0.013	0.02 (0.001, 0.53)	0.018
	Medium	247 (61.8%)	53 (21.4%)	194 (78.6%)	0.41 (0.05, 3.28)	0.398	0.47 (0.05, 4.44)	0.514
	Good	135 (33.8%)	21 (15.6%)	114 (84.4%)	0.60 (0.07, 5.01)	0.640	0.52 (0.05, 5.08)	0.577
	Excellent	10 (2.5%)	1 (10%)	9 (90%)	Reference	-	Reference	-
Religion	Islam	397(99.3%)	79 (19.9%)	318 (80.1%)	Reference	0.090	Reference	0.110
	Other	3 (0.8%)	6 (75%)	1 (33.3%)	0.12 (0.01, 1.39)		0.04 (0.001, 1.98)	
Passing family knowledge course	Yes	330 (82.5%)	57 (17.2%)	273 (82.8%)	Reference	0.002	Reference	0.104
	No	70 (17.5%)	24 (34.2%)	46 (65.8%)	0.40 (0.23, 0.71)		0.56 (0.28, 1.12)	
Participation in sexual health workshop	Yes	77 (19.3%)	9 (11.7%)	68 (88.3%)	2.17 (1.03, 4.56)	0.041	1.31 (0.55, 3.10)	0.531
	No	323 (80.8%)	72 (22.2%)	251 (77.8%)	Reference		Reference	
Subscribe to sexual health social media	Yes	173 (43.3%)	26 (15%)	147 (85%)	1.81 (1.08, 3.03)	0.024	3.23 (1.51, 6.90)	0.002
	No	227 (56.8%)	55 (24.2%)	172 (75.8%)	Reference		Reference	
Use of contraceptive methods	Yes	285 (71.3%)	48 (16.9%)	237 (83.1%)	Reference	0.008	Reference	0.079
	No	115 (28.7%)	33 (28.7%)	82 (71.3%)	0.50 (0.30, 0.84)		0.55 (0.29, 1.07)	
Use of condom	Never	99 (24.8%)	24 (24.2%)	75 (75.8%)	Reference	-	Reference	-
	Usually, not	59 (14.8%)	16 (27.1%)	43 (72.9%)	1.16 (0.56, 2.43)	0.688	0.79 (0.33, 1.86)	0.591
	Sometimes	147 (36.8%)	18 (12.2%)	129 (87.8%)	2.67 (1.25, 5.68)	0.011	1.94 (0.88, 4.29)	0.100
	Always	95 (23.8%)	23 (24.2%)	72 (75.8%)	1.17 (0.56, 2.45)	0.87	0.72 (0.31, 1.65)	0.443
Internet search information source	Yes	258 (64.5%)	61 (23.7%)	197 (76.3%)	Reference	0.024	Reference	0.047
	No	142 (35.5%)	20 (14%)	122 (86%)	0.88 (0.79, 0.98)		1.91 (1.00, 3.64)	
information source of channels and cyberspace	Yes	90 (22.5%)	22 (24.4%)	68 (75.6%)	2.46 (1.40, 4.30)	0.002	3.32 (1.46, 7.56)	0.004
	No	310 (77.5%)	59 (19%)	251 (81%)	Reference		Reference	
Faculty	Medicine	59 (14.8%)	10 (17%)	49 (83%)	Reference	-	Reference	-
	Dentistry	23 (5.8%)	10 (43.4%)	13 (56.6%)	0.27 (0.09, 0.77)	0.015	0.07 (0.10, 0.53)	0.010
	Pharmacy	17 (4.3%)	0 (0%)	17 (100%)	3.27 (0.39, 27.52)	0.277	3.36 (0.15, 73.36)	0.440
	Health	95 (23.8%)	20 (21%)	75 (79%)	0.77 (0.33, 1.77)	0.533	2.07 (0.65,6.60)	0.217
	Nursing and midwifery	107 (26.8%)	17 (15.9%)	90 (84.1%)	1.08 (0.46, 2.54)	0.859	2.59 (0.79, 8.49)	0.115
	Paramedical and New technologies	99 (24.8%)	24 (29.3%)	75 (23.6%)	0.64 (0.28, 1.45)	0.283	1.73 (0.54, 5.48)	0.351
Educational level	Bachelor’s degree	203 (50%)	48 (24%)	155 (76%)	Reference	-	Reference	-
	Master degree	113 (28.2%)	21 (18.6%)	92 (81.4%)	1.31 (0.74, 2.33)	0.361	1.38 (0.66, 2.85)	0.385
	General doctorate	61 (15.3%)	8 (13.1%)	53 (86.9%)	1.42 (0.46, 4.38)	0.543	2.14 (0.51, 8.89)	0.291
	Ph.D	23 (5.8%)	4 (17.3%)	19 (82.7%)	1.98 (0.88, 4.46)	0.100	17.82 (2.29, 138.21)	0.006

Furthermore, students who participated in a sexual health workshop (with percentages of 11.7% for poor SHL and 88.3% for appropriate SHL) demonstrated elevated odds of possessing sexual health literacy (OR = 2.17, 95% CI: 1.03–4.56) relative to those who did not partake in such workshops (with proportions of 22.2% for poor SHL and 77.8% for appropriate SHL). A similar pattern was observed for students subscribed to sexual health-related social media (with percentages of 15% for poor SHL and 85% for appropriate SHL), where higher odds of sexual health literacy were evident (OR = 1.81, 95% CI: 1.08–3.03) compared to those who were not subscribed (with 24.2% for poor SHL and 75.8% for appropriate SHL) (Table 3).

The presence of contraception was significant in sexual health literacy outcomes. Students without contraception (with distribution figures of 28.7% for poor SHL and 71.3% for appropriate SHL) had reduced odds of sexual health literacy (OR = 0.50, 95% CI: 0.30–0.84) compared to those with contraception (with proportions of 16.9% for poor SHL and 83.1% for appropriate SHL). Furthermore, students who occasionally used condoms (with percentages of 12.2% for poor SHL and 87.8% for appropriate SHL) exhibited higher odds of sexual health literacy (OR = 2.67, 95% CI: 1.25–5.68) than those who never used condoms (24.2% for poor SHL and 75.8% for appropriate SHL) (Table 3).

Access to information sources was also influential. Students without the internet as an information source (with distribution percentages of 23.7% for poor SHL and 76.3% for appropriate SHL) demonstrated decreased odds of sexual health literacy (OR = 0.88, 95% CI: 0.79–0.98) compared to those with access to the internet (with 14% for poor SHL and 86% for appropriate SHL). Conversely, students with channels and cyberspace as information sources (with proportions of 24.4% for poor SHL and 75.6% for appropriate SHL) displayed enhanced odds of sexual health literacy (OR = 2.46, 95% CI: 1.40–4.30) compared to those lacking these information sources (with 19% for poor SHL and 81% for appropriate SHL) (Table 3).

Furthermore, a distinction emerged between dentistry students (with distribution percentages of 43.4% for poor SHL and 56.6% for appropriate SHL) and medical students (with 17% for poor SHL and 83% for appropriate SHL). Dentistry students exhibited diminished odds of sexual health literacy (OR = 0.27, 95% CI: 0.09–0.77) in comparison to their medical student counterparts (Table 3).

### Multivariate analysis to assess factors related to sexual health literacy

Students facing poor economic circumstances (with distribution figures of 75% for poor SHL and 25% for appropriate SHL) demonstrated reduced odds of sexual health literacy (OR = 0.03, 95% CI: 0.0–0.55) in contrast to their counterparts with excellent economic status (with proportions of 10% for poor SHL and 90% for appropriate SHL) (Table 3).

Conversely, students who subscribed to sexual health-related social media platforms (with percentages of 15% for poor SHL and 85% for appropriate SHL) exhibited heightened odds of sexual health literacy (OR = 3.27, 95% CI: 1.53–7.01) relative to those who did not engage with such platforms (with 24.2% for poor SHL and 75.8% for appropriate SHL). Similarly, students who accessed information through channels and cyberspace (with distribution proportions of 24.4% for poor SHL and 75.6% for appropriate SHL) displayed elevated odds of sexual health literacy (OR = 3.23, 95% CI: 1.41–7.39) compared to those without access to these sources (with 19% for poor SHL and 81% for appropriate SHL). Students without internet search information source (with distribution proportions of 14% for poor SHL and 86% for appropriate SHL) exhibited heightened odds of sexual health literacy (OR 1.91; 95% CI 1.00- 3.64) compared to those with access to these sources (with 23.7% for poor SHL and 76.3% for appropriate SHL).In terms of academic fields, dentistry students (with distribution percentages of 43.4% for poor SHL and 56.6% for appropriate SHL) exhibited reduced odds of sexual health literacy (OR = 0.07, 95% CI: 0.01–0.52) compared to medical students (with 17% for poor SHL and 83% for appropriate SHL). Ph.D. students (with proportions of 17.3% for poor SHL and 82.7% for appropriate SHL) displayed significantly higher odds of sexual health literacy (OR = 16.93, 95% CI: 2.16–32.70) than bachelor’s students (with 24% for poor SHL and 76% for appropriate SHL) (Table 3).

## Discussion

This study represents a pioneering effort within the University of Iran, delving into sexual health literacy (SHL) levels and their determinants among married students in the medical sciences field. Overall, the study reveals that the SHL and its associated subdomains within the student population are notably adequate. The findings underscore that more than two-thirds of the students exhibit sufficient to high levels of SHL. These findings align not only with prior research conducted in Iran [16, 25] but also with studies from various other nations [5, 17, 30].

Interestingly, the outcomes diverge from those of certain studies. For instance, the results contrast with the findings of Dabiri et al., which showed an insufficient overall level of sexual and reproductive health literacy among young individuals who sought counseling in the Bandar Abbas marriage counseling center in Iran [24]. This inconsistency could be attributed to the potential limited sexual and reproductive health literacy among unmarried youth due to their lack of married life experience. Similarly, the outcomes are incongruent with the study by Vongxay et al., focusing on adolescents aged 15–19 in Laos, where an insufficient overall level of sexual and reproductive health literacy was observed [26]. This could be associated with the lower age range of students in Laos compared to the present study.

Furthermore, the study identified factors influencing SHL among students. Those with poor economic status displayed diminished odds of SHL in comparison to their counterparts with excellent economic status. This resonates with the findings of Barseghian et al., where women of lower socioeconomic status, coupled with a lack of sexual health education programs, were associated with high-risk sexual partnerships, thus affecting SHL [31]. A similar pattern emerged in the study of Jamali et al., where women with dissatisfactory economic statuses exhibited lower SHL compared to their economically content counterparts [16]. This might be attributed to the financial constraints hindering access to sexual health information.

Addressing the social context of young individuals' daily lives, the study highlights the pervasive shame and embarrassment linked with discussing sexual matters. The stigma attached to sexual health topics often acts as a deterrent to seeking information, impeding open discussions and access to healthcare professionals. Encouragingly, students who subscribed to sexual health social media and those with information sources through channels and cyberspace displayed elevated SHL odds. This corresponds with prior research, where the internet emerged as a prominent platform for sexual and reproductive health information [24, 30]. However, divergent information sources were observed in various studies, indicating the diverse ways through which individuals seek such knowledge.

Distinct academic disciplines also demonstrated varying SHL odds. Dentistry students displayed lower SHL odds than medical students. This aligns with an Australian study suggesting that medical and nursing students exhibited higher SHL levels [17]. Ph.D. students exhibited heightened SHL odds compared to bachelor’s students. Consistent results were found in studies conducted by Jamali et al. and Dabiri et al., where education levels were directly correlated with better sexual and reproductive health literacy scores [16, 24]. Education evidently plays a pivotal role in enhancing individuals' understanding, evaluation, and decision-making concerning their health.

The findings, revealing substantial SHL levels among students, are foreseeable. Their engagement with diverse academic courses, including subjects such as physiology and anatomy, contributes to elevated SHL. Additionally, as future medical practitioners, these students carry the responsibility of disseminating health-related information to the public. Thus, the study recommends leveraging these findings to formulate effective educational initiatives that can enhance sexual health literacy among teenagers, young adults, families, teachers, and professors. This can be achieved through strategic planning and collaboration with university administrators to design appropriate educational programs that cater to diverse demographics.

### Study limitations

This study has several limitations. Its cross-sectional design cannot show causality but only generates hypotheses. One limitation of this study was that not all Married Medical Sciences College students in Mazandaran could respond online or did not have access to smartphones, which in itself may lead to limitations in participating in the study. Because this study attempted to collect material in the form of cross-sectional online questionnaires, one of the limitations may be linked to nondifferential information bias due to self-declared answers with possibilities of recall or social desirability biases. In addition, since this was a self-administered questionnaire, students may have been under- or overreporting the information. This may have affected the results to which we are unaware. The confounding risk of bias was low because logistic regression was used to adjust for confounders. Further prospective studies that take into account these pitfalls are necessary to confirm our results.

## Conclusion

In conclusion, this study marks the inaugural exploration of sexual health literacy within the University of Iran's student population. The study's findings indicate that a significant majority of students exhibit satisfactory to high levels of sexual health literacy (SHL). Moreover, certain factors, such as economic status, engagement with sexual health-related social media, information access through digital channels, educational attainment, and academic faculty, are found to be correlated with varying SHL levels. Further investigations building upon this study are warranted to strengthen the validity of the current findings.

Notably, students enrolled in medical sciences colleges will play a pivotal role in public health education, necessitating a heightened awareness in this domain. Leveraging the insights derived from this research, along with sexual health information and collaborative planning involving university administrators, can facilitate the design of targeted educational programs. These initiatives would effectively impart essential knowledge to adolescents, young adults, families, teachers, and professors alike.

### Supplementary Information

**Additional file 1. **Reporting of Observational studies in Epidemiology (STROBE) statement checklist.

**Additional file 2. **Sexual Health Literacy for Iranian Adults (SHELIA) scale.

**Additional file 3. **Sociodemographic and clinical particulars questionnaire.

## Data Availability

The datasets generated during and/or analyzed during the current study are available from the corresponding author on reasonable request.
