# Supplementary material for: Sexual health literacy level and its related factors among married medical sciences college students in an Iranian setting: a web‑based cross‑sectional study
Source: Reprod Health. 2024 Apr 17;21:53. doi: 10.1186/s12978-024-01756-7 (PMC11025204; doi:10.1186/s12978-024-01756-7)
Supplement: Supplementary file 1 — Additional file 1. Reporting of Observational studies in Epidemiology (STROBE) statement checklist. [file 12978_2024_1756_MOESM1_ESM.docx]

Logistic: Variables in the Equation

|  | | B | S.E. | Wald | df | Sig. | OR | 95% C.I.for OR | |
| --- | --- | --- | --- | --- | --- | --- | --- | --- | --- |
|  |  |  |  |  |  |  |  | Lower | Upper |
| Step 1^a^ | Residece(1) | .193 | .541 | .127 | 1 | .721 | 1.213 | .420 | 3.503 |
|  | Economy |  |  | 8.442 | 3 | .038 |  |  |  |
|  | Economy(1) | -3.611 | 1.525 | 5.607 | 1 | .018 | .027 | .001 | .537 |
|  | Economy(2) | -.744 | 1.140 | .426 | 1 | .514 | .475 | .051 | 4.440 |
|  | Economy(3) | -.648 | 1.160 | .312 | 1 | .577 | .523 | .054 | 5.083 |
|  | Religion(1) | -3.017 | 1.889 | 2.550 | 1 | .110 | .049 | .001 | 1.985 |
|  | Famliylesson(1) | -.570 | .350 | 2.647 | 1 | .104 | .565 | .285 | 1.124 |
|  | SexWorkshop(1) | .274 | .438 | .392 | 1 | .531 | 1.316 | .558 | 3.104 |
|  | MembershipinMedia(1) | 1.172 | .387 | 9.160 | 1 | .002 | 3.230 | 1.512 | 6.900 |
|  | Prevention(1) | -.582 | .332 | 3.087 | 1 | .079 | .559 | .292 | 1.070 |
|  | Condom |  |  | 7.182 | 3 | .066 |  |  |  |
|  | Condom(1) | -.235 | .436 | .289 | 1 | .591 | .791 | .336 | 1.860 |
|  | Condom(2) | .665 | .405 | 2.701 | 1 | .100 | 1.944 | .880 | 4.297 |
|  | Condom(3) | -.322 | .420 | .588 | 1 | .443 | .725 | .318 | 1.651 |
|  | net(1) | .650 | .328 | 3.931 | 1 | .047 | 1.916 | 1.007 | 3.643 |
|  | majazi(1) | 1.203 | .419 | 8.246 | 1 | .004 | 3.329 | 1.465 | 7.565 |
|  | reshteRECODE |  |  | 13.207 | 5 | .022 |  |  |  |
|  | reshteRECODE(1) | -2.615 | 1.014 | 6.646 | 1 | .010 | .073 | .010 | .534 |
|  | reshteRECODE(2) | 1.213 | 1.573 | .595 | 1 | .440 | 3.364 | .154 | 73.363 |
|  | reshteRECODE(3) | .729 | .591 | 1.522 | 1 | .217 | 2.073 | .651 | 6.603 |
|  | reshteRECODE(4) | .954 | .605 | 2.488 | 1 | .115 | 2.596 | .793 | 8.496 |
|  | reshteRECODE(5) | .549 | .588 | .870 | 1 | .351 | 1.731 | .546 | 5.485 |
|  | degree_recode_new |  |  | 7.759 | 3 | .051 |  |  |  |
|  | degree_recode_new(1) | .322 | .371 | .754 | 1 | .385 | 1.380 | .667 | 2.852 |
|  | degree_recode_new(2) | 2.881 | 1.045 | 7.600 | 1 | .006 | 17.827 | 2.299 | 138.215 |
|  | degree_recode_new(3) | .765 | .725 | 1.113 | 1 | .291 | 2.148 | .519 | 8.892 |
|  | Constant | -.269 | 1.467 | .034 | 1 | .855 | .764 |  |  |

a. Variable(s) entered on step 1: Residece, Economy, Religion, Famliylesson, SexWorkshop, MembershipinMedia, Prevention, Condom, net, majazi, reshteRECODE, degree_recode_new.

| **Model Summary** | | | |
| --- | --- | --- | --- |
| Step | -2 Log likelihood | Cox & Snell R Square | Nagelkerke R Square |
| 1 | 329.390^a^ | .174 | .273 |
| a. Estimation terminated at iteration number 6 because parameter estimates changed by less than .001. | | | |

**Cox & Snell R Square** and **Nagelkerke R Square**– These are pseudo R-squares.  Logistic regression does not have an equivalent to the R-squared that is found in OLS regression; however, many people have tried to come up with one.  There are a wide variety of pseudo-R-square statistics (these are only two of them).  Because this statistic does not mean what R-squared means in OLS regression (the proportion of variance explained by the predictors), we suggest interpreting this statistic with great caution (ref: <https://stats.oarc.ucla.edu/spss/output/logistic-regression/>

).
