# Supplementary material for: Sexual health literacy level and its related factors among married medical sciences college students in an Iranian setting: a web‑based cross‑sectional study
Source: Reprod Health. 2024 Apr 17;21:53. doi: 10.1186/s12978-024-01756-7 (PMC11025204; doi:10.1186/s12978-024-01756-7)
Supplement: Supplementary file 2 — Additional file 2. Sexual Health Literacy for Iranian Adults (SHELIA) scale. [file 12978_2024_1756_MOESM2_ESM.docx]

1. I can get information about sex education in childhood and adolescence from various sources

strongly agree🞏 agree🞏 neutral🞏 disagree🞏 strongly disagree🞏

2. I can get information about communicable diseases from different source

strongly agree🞏 agree🞏 neutral🞏 disagree🞏 strongly disagree🞏

3. I can get information about sexual problems and disorders in men and women from different sources

strongly agree🞏 agree🞏 neutral🞏 disagree🞏 strongly disagree🞏

4. I can find information about factors affecting sexual relations such as diseases, interpersonal conflicts, marital problems, and complications of the medications taken from various sources

strongly agree🞏 agree🞏 neutral🞏 disagree🞏 strongly disagree🞏

5. I can obtain information on various methods of pregnancy prevention from various sources

strongly agree🞏 agree🞏 neutral🞏 disagree🞏 strongly disagree🞏

6. I can get information about the types of treatment s of Sexual dysfunctions in women and men from different sources

strongly agree🞏 agree🞏 neutral🞏 disagree🞏 strongly disagree🞏

7. I can get information about sex in old age from various sources

strongly agree🞏 agree🞏 neutral🞏 disagree🞏 strongly disagree🞏

8. It is easy for me to read about sex education (books, booklets, pamphlets, educational and promotional brochures) during childhood and adolescence

strongly agree🞏 agree🞏 neutral🞏 disagree🞏 strongly disagree🞏

9. It is easy for me to read educational materials related to couples' sexual relations and the factors that affect them

strongly agree🞏 agree🞏 neutral🞏 disagree🞏 strongly disagree🞏

10. It is easy for me to read educational materials related to sexually transmitted diseases

strongly agree🞏 agree🞏 neutral🞏 disagree🞏 strongly disagree🞏

11. It is easy for me to read educational materials related to various methods of contraception

strongly agree🞏 agree🞏 neutral🞏 disagree🞏 strongly disagree🞏

12. It is easy for me to read educational materials related to the treatment of sexual dysfunctions in men and women

strongly agree🞏 agree🞏 neutral🞏 disagree🞏 strongly disagree🞏

13. It is easy for me to read educational materials related to couples' sexual relations in old age

strongly agree🞏 agree🞏 neutral🞏 disagree🞏 strongly disagree🞏

14. If I have a sexual problem and consult a specialist or counselor, it is easy for me to read the written instructions given about my problem

strongly agree🞏 agree🞏 neutral🞏 disagree🞏 strongly disagree🞏

15. If I have a sexual problem and refer to a specialist or counselor, it is easy for me to read the guidelines (preparation before tests, pelvic exams, ultrasound, or urogenital examinations)

strongly agree🞏 agree🞏 neutral🞏 disagree🞏 strongly disagree🞏

16. I can understand the issues related to the sexual education and training of children and adolescents

strongly agree🞏 agree🞏 neutral🞏 disagree🞏 strongly disagree🞏

17. I can understand the issues related to improving the couple's sexual relations

strongly agree🞏 agree🞏 neutral🞏 disagree🞏 strongly disagree🞏

18. I understand the issues related to the prevention and treatment of sexually transmitted diseases

strongly agree🞏 agree🞏 neutral🞏 disagree🞏 strongly disagree🞏

19. I understand the issues related to improving sexual relations in old age

strongly agree🞏 agree🞏 neutral🞏 disagree🞏 strongly disagree🞏

20. In case of sexual problems and referring to a specialist and counselor, I will understand the explanations that she gives me about my problem

strongly agree🞏 agree🞏 neutral🞏 disagree🞏 strongly disagree🞏

21. In case of sexual problems and referral, I will understand the meaning and concept of the contents written in the relevant forms such as patient admission form, consent, and file formation

strongly agree🞏 agree🞏 neutral🞏 disagree🞏 strongly disagree🞏

22. In case of sexual problems and referral for treatment, I will understand the meaning and concept of the symptoms and the contents written on the signboards in the relevant clinics

strongly agree🞏 agree🞏 neutral🞏 disagree🞏 strongly disagree🞏

23. In case of sexual problems and prescribing medicine, I will understand how to use the medicine that is written on the package

strongly agree🞏 agree🞏 neutral🞏 disagree🞏 strongly disagree🞏

24. In case of sexual problems and receiving treatment or advice, I will understand its advantages and disadvantages

strongly agree🞏 agree🞏 neutral🞏 disagree🞏 strongly disagree🞏

25. I realize the harms of doing things like watching immoral movies, drinking alcohol, smoking, and having extra-marital sex on my sexual health

strongly agree🞏 agree🞏 neutral🞏 disagree🞏 strongly disagree🞏

26. I believe in the accuracy of the information I get about sex through various sources

strongly agree🞏 agree🞏 neutral🞏 disagree🞏 strongly disagree🞏

27. I can evaluate the accuracy of sexual health information provided on the Internet

strongly agree🞏 agree🞏 neutral🞏 disagree🞏 strongly disagree🞏

28. I can evaluate the accuracy of sexual health information provided by television, radio, and satellite networks

strongly agree🞏 agree🞏 neutral🞏 disagree🞏 strongly disagree🞏

29. I can pass on the information I have learned about sexual health to others correctly

strongly agree🞏 agree🞏 neutral🞏 disagree🞏 strongly disagree🞏

30. As soon as I realize a sexual problem or disorder, I know where or to whom I should go

strongly agree🞏 agree🞏 neutral🞏 disagree🞏 strongly disagree🞏

31. If I have a sexual problem, I follow the treatment recommendations such as taking medicine for one hour before sexual intercourse

strongly agree🞏 agree🞏 neutral🞏 disagree🞏 strongly disagree🞏

32. In the case of a sexual problem, I will not discontinue the techniques recommended for resolving my sexual problem without the counselor's permission, even if my sexual problem is gone

strongly agree🞏 agree🞏 neutral🞏 disagree🞏 strongly disagree🞏

33. If my spouse has a sexual problem, I will go with him for sexual counseling

strongly agree🞏 agree🞏 neutral🞏 disagree🞏 strongly disagree🞏

34. Even if I do not have a sexual problem, I go to a sex counselor to get an education and improve the quality of sex with my spouse

strongly agree🞏 agree🞏 neutral🞏 disagree🞏 strongly disagree🞏

35. If I have any questions about my sexual health, I will ask the relevant counselor

strongly agree🞏 agree🞏 neutral🞏 disagree🞏 strongly disagree🞏

36. I take care of my sexual health in any situation

strongly agree🞏 agree🞏 neutral🞏 disagree🞏 strongly disagree🞏

37. In any situation, I take care of the quality of sex with my spouse

strongly agree🞏 agree🞏 neutral🞏 disagree🞏 strongly disagree🞏

38. I avoid asking for sex if my spouse is not physically and mentally ready (sexual coercion)

strongly agree🞏 agree🞏 neutral🞏 disagree🞏 strongly disagree🞏

39. When having sex with my spouse, I pay attention to human values such as maintaining dignity, mutual respect, observing moral standards, and so on

strongly agree🞏 agree🞏 neutral🞏 disagree🞏 strongly disagree🞏

40. I usually use the information I get from various sources about sexual health

strongly agree🞏 agree🞏 neutral🞏 disagree🞏 strongly disagree🞏
