# Supplementary material for: Sexual health literacy level and its related factors among married medical sciences college students in an Iranian setting: a web‑based cross‑sectional study
Source: Reprod Health. 2024 Apr 17;21:53. doi: 10.1186/s12978-024-01756-7 (PMC11025204; doi:10.1186/s12978-024-01756-7)
Supplement: Supplementary file 3 — Additional file 3. Sociodemographic and clinical particulars questionnaire. [file 12978_2024_1756_MOESM3_ESM.docx]

| Sociodemographic and clinical particulars | |
| --- | --- |
| Age range | 15-25 |
|  | 26-35 |
|  | 36-45 |
|  | >45 |
| Gender | Female |
|  | Male |
| Living area | City |
|  | Urban |
| Educational level | Bachelor’s degree |
|  | Master degree |
|  | General doctorate |
|  | Ph.D. |
| Faculty | Medicine |
|  | Dentistry |
|  | Pharmacy |
|  | Health |
|  | Nursing and midwifery |
|  | Paramedical |
|  | New technologies |
| Spouse's education level | High school and diploma |
|  | Bachelor’s degree |
|  | Master degree |
|  | General doctorate |
|  | Ph.D. |
| Religion | Islam |
|  | Other* |
| job | With a fixed salary |
|  | No fixed salary |
|  | Student |
| Duration of marriage (year) | Less than 1 |
|  | 1-3 |
|  | 3-5 |
|  | More than 5 |
| Sexual Information Source | Friends |
|  | Parents |
|  | Sister and brother |
|  | Relatives |
|  | Internet |
|  | Channels and cyberspace |
|  | Media |
|  | Health care providers |
| Passing family knowledge course | Yes |
|  | No |
| Economic status | Weak |
|  | Medium |
|  | Good |
|  | Excellent |
| Student's residence | Dormitory |
|  | Private house |
| Participation in sexual health workshop | Yes |
|  | No |
| Premarital sexual experience | Yes |
|  | No |
| Subscribe to social media** for sexual health | Yes |
|  | No |
| Media and Internet access | Yes |
|  | No |
| Use of contraceptive methods | Yes |
|  | No |
| Type of contraception | Natural methods |
|  | Condom |
|  | Tablet |
|  | Injection |
|  | Intrauterine devices |
|  | Surgery |
|  | None |
| Use of condom | Always |
|  | Sometimes |
|  | Usually, not |
|  | Never |
| Abortion | Never |
|  | Once |
|  | More than once |
| History of sexual abuse | Yes |
|  | No |
| History of sexually transmitted diseases | Yes |
|  | No |
